# Methods of Microencapsulation of Vegetable Oil: Principles, Stability and Applications - A Minireview

**DOI:** 10.17113/ftb.60.03.22.7329

**Published:** 2022-09

**Authors:** Luana Carvalho da Silva, Rachel Menezes Castelo, Huai N. Cheng, Atanu Biswas, Roselayne Ferro Furtado, Carlucio Roberto Alves

**Affiliations:** 1 State University of Ceará, Science and Technology Center, 60.714-903, Fortaleza, CE, Brazil; 2 USDA Agricultural Research Service, Southern Regional Research Center, 1100 Robert E. Lee Blvd., New Orleans, LA 70124, USA; 3 USDA Agricultural Research Service, National Center for Agricultural Utilization Research, 1815 North University Street, Peoria, IL 61604, USA; 4 Embrapa Agroindústria Tropical, ZIP code: 60.511-110, Fortaleza, CE, Brazil

**Keywords:** microparticles, vegetable oil, oxidative stability, fatty acids, controlled release

## Abstract

In addition to being used in food, fuel and lubricants, vegetable oils are promising in many other applications such as food additives, nutritional supplements, cosmetics and biomedicine; however, their low oxidative stability can limit their use. Microencapsulation is a well-established method for the preservation of oil against degradation, controlled release of active ingredients, protection against external factors during storage, and enhanced durability. In this article, microencapsulation methods for vegetable oil are reviewed, including physical methods (spray-drying and freeze-drying), physicochemical methods (complex coacervation, ionic gelation and electrostatic layer-by-layer deposition), and chemical methods (interfacial/*in situ* polymerization). This article also provides information on the principles, parameters, advantages, disadvantages and applications of these methods.

## INTRODUCTION

Vegetable oils are gaining a lot of attention in commercial development these days because of their availability, low price, biodegradability and mild environmental impact. They contain, as their main components, triglyceride esters of glycerol with three long-chain fatty acids (*1*, *2*). The fatty acids can be the same or different in terms of the hydrocarbon chains formed from 10 to 22 carbon atoms. In addition, the location and the number of double bonds in the fatty acid chains cause these compounds to exhibit different physical and chemical properties (*2*). One of the most important parameters that influence lipid oxidation is the degree of unsaturation of the fatty acids (*3*). Oxidation caused by several external factors such as temperature, light, presence of oxygen and humidity can lead to the formation of unpleasant flavours and odours, reduction in the product shelf life and generation of free radicals, which can have negative physiological effects on the body (*4*). The reason to use microencapsulation methods is to protect the oil against these external factors, along with the possibilities of masking its odours and flavours and providing release control.

Microencapsulation is a technique in which one or more substances (*e.g.* a core substance, an active material or a separate phase in a mixture) are surrounded or immobilized by one or more materials (*e.g.* a shell, polymer matrix, support or wall material) and protected from biotic and abiotic factors (*5*). It is an effective technology to protect fatty acids and associated vitamins from oxidative degradation (*6*). The shell and core characteristics are important factors that play a critical role in determining the encapsulation efficiency, core stability and other microencapsulation physicochemical characteristics (*7*).

Several methods of microencapsulation (*8*) can be divided into physical methods (spray-drying and freeze-drying), physicochemical methods (complex coacervation, ionic gelation and electrostatic layer-by-layer deposition), and chemical methods (interfacial polymerization and *in situ* polymerization). The choice of a microencapsulation method depends on the active material and encapsulating matrix, as well as the prospective application area (Table 1 (*9*-*27*)).

**Table 1:** Recent representative studies of the methods, core substances, encapsulating matrices and (suggested) applications for microencapsulated vegetable oil

Method	Vegetable oil	Encapsulating matrix	Application	Reference
Spray-drying	Linseed oil	Different combinations of maltodextrin, gum arabic, whey protein and methyl cellulose	Food (bread)	(*9*)
	Linseed oil	Modified starch	Food	(*10*)
	Green coffee oil	Different combinations of modified starch, gum arabic and maltodextrin	Food	(*11*)
	Green coffee oil	Gum arabic	Cosmetics	(*12*)
	Cress seed oil	Whey protein	Food (biscuit)	(*13*)
Freeze-drying	Olive oil	Different combinations of maltodextrin, carboxymethylcellulose and lecithin	Food	(*14*)
	Walnut oil	Different combinations of sodium caseinate, maltodextrin, lecithin and carboxymethylcellulose	Food	(*15*)
Complex coacervation	Corn oil	Xylitol and gelatin	Suggested application in food	(*16*)
	Palm oil	Chitosan/xanthan and chitosan/pectin	Food (yogurt and bread)	(*17*)
	Pequi oil	Cashew gum/chitosan	Cosmetics	(*18*)
	Pomegranate seed oil	Whey protein/gum arabic	Food	(*19*)
	Green coffee oil	Cashew gum/gelatin	Food (juice)	(*20*)
Ionic gelation	Chia oil	Sodium alginate and calcium chloride	Food (hamburger)	(*21*, *22*)
Electrostatic layer-by-layer deposition	Linseed oil	Bovine serum albumin (emulsifier), poly-l-arginine and dextran sulfate	Food	(*23*)
	Sunflower oil	Bovine serum albumin, poly(sodium 4-styrenesulfonate) and poly(allylamine hydrochloride)	Food	(*24*)
	Green coffee oil	Lecithin and chitosan	Cosmetics	(*25*)
	Chia oil	Modified sunflower lecithin, chitosan and maltodextrin	Food	(*26*)
Polymeriza-tion *in situ*	Neem oil	Phenol formaldehyde	Insecticide	(*27*)

This review aims to present the methods commonly used in the microencapsulation of vegetable oils. The principles of each method, operating parameters, advantages and disadvantages will first be presented (Table 2). Then, representative studies will be discussed, highlighting their main objectives and possible applications.

**Table 2:** Advantages and disadvantages of physical, physicochemical and chemical methods commonly used for microencapsulation of vegetable oil

Method	Advantage	Disadvantage
Spray-drying	Availability of equipment in industry; potentially large-scale production and simple equipment; high efficiency and low process cost	Limited number of available wall materials that have good solubility in water; low percentage of active molecules being carried
Freeze-drying	Very good rehydration behavior of the powdered product; high product quality	Long drying time; low temperature and high vacuum; high operational cost
Complex coacervation	More moderate reaction conditions during processing; lower equipment cost; greater loading capacity	Optimization very time-consuming and laborious; operational parameters can affect a series of physical and chemical properties
Ionic gelation	Relatively low cost; does not require specialized equipment, high temperature or an organic solvent	Gelling bath, complex nature of the formulation; time consuming and low scale reproducibility
Electrostatic layer-by-layer deposition	Protecting emulsion droplets from oxidation or lipid aggregation; controlling or releasing active materials; improving stability against environmental agents due to the thicker interfacial layers	Limitation of wide commercialization due to the high cost generated by the precise control over the composition of the system

## MICROENCAPSULATION METHODS FOR VEGETABLE OIL

### Physical methods

In physical methods, the microcapsule wall is mechanically applied or condensed around the microcapsule core. Some of these methods are widely used in modern food industry due to their ease of preparation and economic benefit. They may also be used in combination with physicochemical and chemical methods such as drying after microencapsulation.

#### Spray-drying

**Fig. S1:**
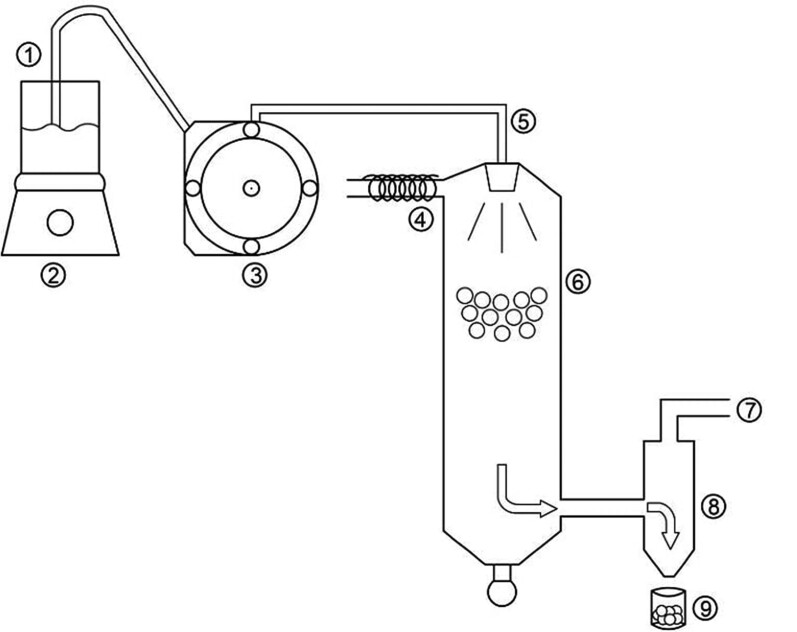
Representative scheme of the spray-drying microencapsulation process, where: 1=feeder, 2=magnetic stirrer, 3=peristaltic pump, 4=hot air/gas inlet, 5=atomizer nozzle, 6=drying chamber, 7=gas extractor, 8=cyclone, and 9=particle collector

Principles. Spray-drying is one of the commonest methods used in microencapsulation of vegetable oil. The method consists of a process capable of transforming solutions, suspensions or emulsions into a solid product. The spray-drying process (Fig. S1) can be defined as an operation in which a liquid stream pumped into an atomizer is constantly divided into very fine droplets inside the drying chamber. A polymer, which serves as the encapsulant, is usually dissolved in the solution or in the continuous phase of a suspension or emulsion. In the drying chamber, the fine droplets come into contact with hot air, which by convection provides energy for heating and vaporizes most of the solvent present in the droplets, forming dust particles. These are separated from the drying gas using a cyclone or a filter bag (*28*).

Physical properties of emulsions and atomization parameters are important factors used to define the droplet formation, particle size and other parameters such as retention/encapsulation efficiency, physicochemical properties, yield and storage stability (*4*, *11*). For oil encapsulation, the first step is to emulsify the core ingredient in a polymer solution. Different types of emulsion, such as single- or multilayered oil-in-water (o/w) emulsions, have been employed to entrap oils (*4*).

Atomization parameters are related to the spray-drying equipment: inlet/outlet temperature, feeding flow, atomizer gas flow, atomizer gas type and nozzle size (*29*). Laboratory-scale spray-dryer usually has settings that allow the operator to vary the particle properties. The process can be modified in terms of its cycle mode, atomizer type and airflow rate. However, this modularity becomes limited on an industrial scale due to financial and technological difficulties. For example, while a variation in atomizer or airflow is viable on an industrial scale, a change of the cyclone or the drying chamber geometry can be very expensive (*30*).

This process has some advantages over other methods, such as the availability of the equipment in industry, the possibility of using a wide variety of encapsulating materials, potentially large-scale production, simple equipment, high efficiency and low process cost (*31*). The main limitation of spray-drying in microencapsulation is the limited number of wall materials available, and they must have good solubility in water. Another disadvantage of spray-drying is that the end-product is often a fine microcapsule powder that needs further processing, *e.g.* to remove agglomeration (*29*). Moreover, this technique provides only a limited payload; the percentage of active molecules carried is generally 10–30%.

Stability. A large number of vegetable oils have been microencapsulated using the spray-drying method. Bae and Lee (*32*) microencapsulated avocado oil in whey protein and maltodextrin matrix and obtained good results in improving the oil oxidative stability in different matrix concentrations. Similar studies have been reported that emphasize the improvements in the oxidative stability of microencapsulated oil by spray-drying (*33*). In particular, the results obtained by González *et al.* (*33*) showed that the microencapsulation of chia oil by spray-drying with different wall materials provided twice as strong protection for all other samples, under accelerated oxidative conditions.

Gomes and Kurozawa (*34*) evaluated the potential antioxidant use of hydrolyzed rice protein in the microencapsulation of linseed oil and reported a reduction in its lipid oxidation during storage due to the greater antioxidant capacity of the protein hydrolysate. Oliveira *et al*. (*35*) evaluated the effect of a partial replacement of whey protein isolate by maltodextrin and inulin on the pequi oil microcapsules and also reported on the degradation of bioactive compounds. These studies showed an improvement in the stability of the microencapsulated oil, which may also be related to the choice of wall material used mainly due to its antioxidant capacity.

Applications. Nosari *et al*. (*12*) observed the antioxidant activity of microencapsulated green coffee oil in gum arabic matrix under the effect of light, heat and oxygen and verified better stability results than with non-encapsulated oil, even better than with the addition of α-tocopherol, an antioxidant widely used in the cosmetic and food industries. Gallardo *et al.* (*9*) microencapsulated linseed oil, rich in ω-3 fatty acids, in gum arabic matrix and applied it to bread. The fortified bread showed a similar appearance to the control bread without microcapsules, but the α-linolenic acid content was significantly reduced. Umesha *et al.* (*13*) enriched cookies with watercress seed oil, rich in α-linolenic acid, microencapsulated in whey protein. The oxidation rate of α-linolenic acid was higher in cookies supplemented with pure oil than in cookies with microencapsulated oil, indicating that encapsulation reduced the oxidation of α-linolenic acid in the cookies.

### Freeze-drying

Principles. Freeze-drying or lyophilization is one of the microencapsulation methods most commonly used for thermosensitive molecules, being a good alternative to the spray-drying (*36*). Thus, it represents a useful approach for the preparation of products containing oil. Microparticles with high resistance to thermal and oxidative degradation and good encapsulation efficiency have been produced (*33*, *37*). During the freeze-drying, the material temperature is reduced below its freezing point and the water is removed by sublimation at pressures below that of the triple point of water (*38*).

Freeze-drying can be divided into three stages: freezing (solidification), primary drying (ice sublimation) and secondary drying (thawed water desorption) (*39*, *40*). During the freezing stage, most of the water is converted into a solid, where ice crystal networks are formed. It is at this stage that the morphology of materials, the size and the size distribution of the ice crystals are determined, which in turn influence several critical parameters, such as dry product resistance, primary and secondary drying rates, extent of product crystallinity, surface area, and dry product reconstitutability (*39*-*41*).

Primary drying is the second stage of the freeze-drying process, and it is closely related to the preceding freezing stage (*42*). The sublimation of the ice starts from the top surface of the sample and continues to the bottom. For samples that are quickly frozen and form small ice crystals that prevent the mass transfer of sublimated water vapor through the dry layer, the primary drying can take a long time. On the other hand, slow freezing forms large ice crystals that facilitate the movement of water vapor (the mass transfer rate being high) and, as a result, the primary drying time is reduced (*43*). During primary drying, the free frozen water is removed by converting the ice to vapor (sublimation). The drying process depends on the shelf temperature and the chamber pressure, and the appropriate choice of these two parameters can shorten the process time (*42*).

The last stage of freeze-drying is secondary drying, where the water absorbed from the product is removed. This is the water that did not form ice during freezing and did not sublimate (*39*). During secondary drying, the shelf temperature increases even more, while the pressure is kept constant or, in some cases, is lower than that of primary drying (*42*). The sorbed water that remains in the solute matrix is then further reduced by desorption (*44*).

The main advantage of using freeze-drying is that porous structures are formed by the sublimation of ice crystals, which leads to a good rehydration behavior of the powdered product. The freeze-dried particles generally have a high quality, but the long drying times, batch production, low temperatures, high vacuum and high operational cost limit the usage of the freeze-drying technique (*44*).

Stability. Some recent studies have used freeze-drying as a microencapsulation method for vegetable oil. Aksoylu Özbek and Günç Ergönül (*45*) evaluated the interaction effect of whey protein, maltodextrin and gum arabic in microencapsulation of pumpkin seed oil in relation to emulsion stability, encapsulation efficiency, solubility, wetting time, total polyunsaturated and saturated fatty acid content. Comunian *et al*. (*46*) studied the co-encapsulation of pequi and buriti oil using whey protein in order to obtain better carotenoid retention and oxidative stability and compared the emulsion with and without the freeze-drying process. The freeze-dried samples showed the best carotenoid retention and oxidative stability, indicating that emulsified and then freeze-dried oil can serve as effective carrier of bioactive compounds.

Souza *et al*. (*47*) microencapsulated the chia oil, rich in polyunsaturated fatty acids (omega-3 and omega-6), in order to increase its stability during processing and storage. Differential scanning calorimetry showed an increase in the oxidative stability of the encapsulated oil, which may indicate that such microparticles are suitable to formulate food products where long shelf life is needed or when heating is applied during production such as in baked products.

Applications. Calvo *et al.* (*14*) evaluated the influence of microencapsulation on the chemical composition of extra virgin olive oil and its oxidative stability and concluded that it prolonged its shelf life. Microencapsulated oil in protein-based microcapsules was found to be unaltered for 9 to 11 months. It was concluded that the presence of protein constituents in the microcapsule wall material extended the shelf life of the microencapsulated olive oil. Calvo *et al.* (*15*) studied the *in vitro* digestibility of microencapsulated walnut oil using different types of microcapsules and the availability of ω-3, ω-6 fatty acids and tocopherols after microcapsule digestion. It was found that protein-based microcapsules were highly digestible; 90% of the encapsulated oil was released from the capsule after *in vitro* digestion. Both studies suggest the application of the encapsulated materials in food, providing a viable procedure to add highly unsaturated and tocopherol-enriched oil to processed foods, and ensuring its bioavailability without altering the organoleptic properties.

### Physicochemical methods

The physicochemical methods entail the electrostatic interactions generated by the specific components of the system, which can be varied by mechanical agitation, pH and temperature changes, leading to the formation of a solid and stable particle. Droplet properties such as component composition, surface charge, thickness and responsiveness to environmental stresses can be optimized by carefully controlling system composition and preparation conditions. After the physicochemical treatment, drying methods such as spray-drying or freeze-drying are used in order to improve the application and storage of the particles and a better quality for the final product.

#### Complex coacervation

Principles. Coacervation is a term used in colloidal chemistry to denote the process of formation of an associative phase induced by the environment modification (pH, ionic strength, temperature and solubility) under controlled conditions (*6*, *48*). Complex coacervation is a phenomenon of liquid-liquid phase separation that occurs between oppositely charged polymers through electrostatic interactions (*49*). Complex coacervation functions in microencapsulation by creating a barrier around the active material and preventing active compounds from interacting physically and/or chemically with the external environment (*50*). This technique of microencapsulation has been particularly successful in stabilizing unsaturated lipids and providing a product with a consistent sensory shelf life (*51*).

**Fig. S2:**
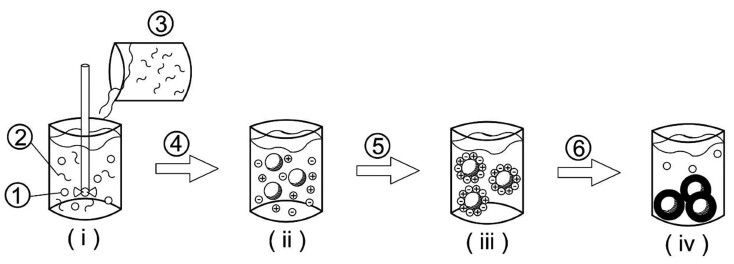
Formation of the complex coacervation involving four steps: (*i*) preparing an aqueous solution of two or more polymers and mixing the hydrophobic phase with the aqueous solution of a polymer, often a protein solution, and homogenizing the resulting mixture in order to produce a stable emulsion, where: 1=oil dispersed in the emulsion, 2=first solubilized polymer, 3=second solubilized polymer, (*ii*) pH change, where each polymer assumes its respective effective charges, where: 4=pH adjustment, (*iii*) change in temperature to a certain value necessary to induce coacervation and phase separation, where: 5=refrigeration, and (*iv*) polymer hardening using high temperature, desolvation agent or crosslinker: 6=precipitation of coacervates

Complex coacervation (Fig. S2) comprises a three-phase system that involves the solvent, the active material and the coating material. In general, this process for emulsions involves four steps: (*i*) preparing an aqueous solution of two or more polymers and mixing the hydrophobic phase with the aqueous solution of a polymer, often a protein solution, and homogenizing the resulting mixture, in order to produce a stable emulsion, (*ii*) pH change, where each polymer assumes its respective effective charges, (*iii*) change in temperature to a certain value necessary to induce coacervation and phase separation, and (*iv*) polymer hardening using high temperature, desolvation agent or crosslinker (*48*).

Several operational parameters influence the complexation of the encapsulation procedure through electrostatic interactions (ionic strength), pH, composition of the encapsulating matrix, matrix concentration, charge distribution, homogenization, macromolecules solubility and other molecular properties related to physical and chemical conditions of the solution. Therefore, a greater understanding of these parameters is fundamental for a better coacervation and its more efficient application (*49*, *52*, *53*). Although the electrostatic force between oppositely charged macromolecules is the main driving force, van der Walls intermolecular forces and hydrophobic interactions in proteins also affect the complex coacervation process (*6*).

After the coacervate formation, the microcapsules can be dried in order to improve storage and increase their shelf life. Among the drying methods, the most commonly used are spray- (*54*, *55*) and freeze-drying (*50*, *56*-*58*). The choice of the drying method will depend on the nature of the encapsulating matrix and the active material.

Complex coacervation is a classic method of microencapsulation with great advantages, including more moderate reaction conditions during processing, lower equipment cost and greater loading capacity (*5*). However, there are some limitations to the utilization of complex coacervation for the encapsulation of less stable substances such as oil. One disadvantage is that the process demands a narrow pH and ionic strength range in which the coacervates are stable. In general, it is often necessary to cross-link soon after coacervate formation to prevent dissociation (*59*). Other disadvantages are related to the optimization process that can be time-consuming and laborious, as each of the operational parameters can affect a series of physical and chemical properties of the flow system during complex coacervation (*52*).

Stability. Complex coacervation is a highly recommended method for the microencapsulation of lipophilic substances. Several studies use this method to encapsulate vegetable oils. The thickness of the outer shell can be higher using complex coacervation than using other methods, leading to improved oxidative and sensory stability (*59*). Soares *et al.* (*58*) microencapsulated the sacha inchi (*Plukenetia volubilis* L.) oil, rich in ω-3, in ovalbumin matrix and sodium alginate, obtaining microcapsules with good thermal behaviour and protection of the bioactive compounds in the oil. Linseed oil, a rich source of ω-3 fatty acids, was microencapsulated by Kaushik *et al.* (*60*) in a matrix formed by complex coacervation of flaxseed protein isolate and flaxseed gum. This matrix was cross-linked with glutaraldehyde and spray-dried or freeze-dried, and better results for oxidative stability were obtained than with the non-encapsulated oil. Lemos *et al.* (*56*) investigated the influence of hydrodynamic conditions in the buriti oil coacervation, rich in carotenoids, using gelatin/alginate as matrix and identified the agitation speed as having a strong influence on the microcapsule size. Justi *et al.* (*61*) studied the pequi oil microencapsulation using gelatin and gum arabic as encapsulating matrices in order to improve the oil stability. In that study, the influence of temperature, agitation speed and core material on oil coacervation was evaluated in the preservation of carotenoids present in the oil. Timilsena *et al*. (*7*) obtained chia seed oil microcapsules in chia seed protein/chia seed gum matrix to improve the oil oxidative stability.

A few studies optimized the microcapsule formation parameters by complex coacervation. Devi *et al.* (*62*) microencapsulated the olive oil in gelatin/sodium alginate matrix and optimized the proportions of the biopolymers and pH. Silva *et al.* (*18*) studied cashew gum/chitosan for pequi oil microencapsulation and optimized the formation parameters for coacervates (*viz*. biopolymer charge, pH and matrix ratios). A similar study was conducted by Nascimento *et al.* (*63*) with the cashew gum/gelatin matrix.

Applications. Rutz *et al*. (*17*) studied the palm oil microencapsulation, rich in carotenoids, in chitosan/xanthan and chitosan/pectin matrices followed by spray-drying and freeze-drying. Freeze-dried microparticles resulted in lower carotenoid loss, and higher yield and encapsulation efficiency, and chitosan/pectin microparticles showed a better release profile. After the application of microparticles in food (bread and yogurt), the release of the carotenoids was lower, and the released compounds were not degraded. Chitosan/xanthan microparticles have the best potential to be successfully applied in the food industry, particularly in yogurt preparations. Oliveira *et al.* (*20*) explored the complexation of cashew gum and gelatin to encapsulate green coffee oil, rich in cafestol and kahweol, for use as ingredient in fruit juice. The beverage with added capsules showed good sensory quality when compared to the control formulation, promoting a diterpene-rich drink with good rheological and sensory properties.

#### Ionic gelation

Principles. Ionic or ionotropic gelation is a microencapsulation process that starts from an aqueous polymeric solution, where ions with a low molecular mass interact with oppositely charged polyelectrolytes forming an insoluble gel (*64*). It can be used to encapsulate hydrophilic or hydrophobic compounds (*65*). The active material to be encapsulated is dissolved/dispersed or emulsified in the polymeric solution. The drops of polymer solution that reach the ionic solution stimulate the formation of spherical gel structures, which contain the active material dispersed throughout the polymer matrix (*66*, *67*).

This technique was adopted for natural polysaccharides to produce biocompatible and biodegradable products (*68*). Sodium alginate is the most used biopolymer for ionic gelation due to its gelling property and chemical structure. Having carboxylic groups, alginates readily form gels in the presence of calcium ions or other divalent or trivalent cations, and a high guluronate content in alginate can produce stronger gels (*69*).

This technique is simple, easy to encapsulate substances, relatively low cost, and does not require specialized equipment, high temperature or an organic solvent (*5*, *65*, *66*). In addition, the ionic gelation technique has several more advantages, such as the use of aqueous solutions, small particle size, better control of the particle size through variations in the precursor concentration, and the possibility of encapsulating a wide variety of substances (*70*, *71*). The ionic gelation technique has as disadvantages the need for a gelling bath, the complex nature of the formulation, the time-consuming process, and low-scale reproducibility, which can be improved by using the nozzle vibration technique (NVT), which improves uniformity and scalability. The process can be conducted under mild, non-toxic conditions to preserve the integrity even of extremely labile bioactive compounds (*68*).

Ionic gelation can be used combined with NVT that is featured in some equipment. The NVT causes the formation of droplets of the same size by superimposing vibrations on a fluid jet through a precisely drilled nozzle. The selected vibration frequency determines the number of produced droplets (*72*). This technology has gained significant interest for its ability to produce reproducible microspheres with defined sizes, thereby generating uniform and monodisperse particles (*73*). For the optimization of dispersed droplets, some parameters must be well defined, such as feed flow rate, vibration frequency, vibration amplitude and electrode voltage (*74*, *75*).

Stability. This technique is also applicable for microencapsulation of vegetable oils. Because it does not entail thermal stress, some recent studies used this technique to improve the oxidative stability of the encapsulated active ingredients. Menin *et al.* (*68*) employed ionic gelation combined with NVT and obtained particles of flaxseed oil, using pectin as wall material, achieving oxidative stability up to 13 times greater than of the non-encapsulated oil. Sathasivam *et al.* (*76*) used ionic gelation/NVT in the microencapsulation of red palm oil with carboxymethylcellulose matrix and obtained capsules with good thermal stability. Oxidative stability test confirmed minor degradation of oil in the beads. An initial increase in peroxide value of the encapsulated oil was associated with the oxidation of the surface oil which was in contact with oxygen at the highest temperature. Later, the peroxide value of the oil-loaded beads did not increase much and was lower than of the control, showing the protection against oxidation.

Applications. Heck *et al.* (*21*) used ionic gelation to microencapsulate the chia oil enriched with rosemary that was targeted to replace 50% fat in hamburgers. The hamburgers produced with microparticles of chia oil enriched with rosemary showed greater oxidative stability, especially after cooking. In addition, the incorporation of rosemary antioxidants into chia oil reduced the sensory defects caused by lipid oxidation. Complementing the previous studies, Heck *et al.* (*22*) evaluated the volatile compounds and sensory properties of frozen hamburgers during storage, which resulted in a decrease in lipid and protein oxidation volatiles and an increase in terpenes at the beginning (day 1) and at the end of storage (day 120), before and after cooking.

#### Electrostatic layer-by-layer deposition

**Fig. S3:**
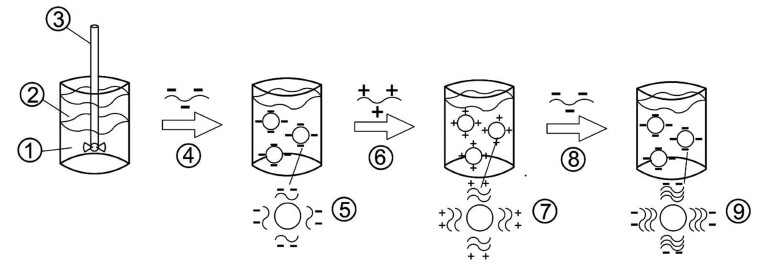
Production steps for the electrostatic layer-by-layer deposition, where: 1=water, 2=oil, 3=agitation, 4=emulsifier, 5=primary emulsion, 6=polymer I, 7=secondary emulsion, 8=polymer II, and 9=tertiary emulsion

Principles. In electrostatic layer-by-layer deposition (Fig. S3), an ionic emulsifier that quickly adsorbs on the surface of lipid droplets during homogenization is used to produce a primary emulsion containing small droplets; then, an oppositely charged polyelectrolyte is added to the system which adsorbs on the droplet surfaces and produces a secondary emulsion containing droplets coated with a two-layer interface. This procedure can be repeated to form oil droplets coated with interfaces containing three or more layers (*77*, *78*). The deposited polymeric coatings allow substances to withstand various environmental stresses, such as pH, ionic strength, freezing and heating and show improved performance relative to uncoated ones (*79*, *80*).

Layer-by-layer technology is simple, versatile and may be used to develop multilayer coatings when needed (*81*). The ionic characteristic (electric charge) must be evaluated as a function of the pH between the emulsifier and each biopolymer used in the system to determine the conditions for best electrostatic attraction between them (*77*). The effectiveness can often be optimized by controlling the number, type and sequence of biopolymer layers used to coat the lipid droplets, as this allows the control of the thickness, composition, charge, permeability and integrity of the interfaces (*82*).

Drying methods are recommended after the electrostatic layer-by-layer deposition process to transform the material into powder, with the objective of improving handling and storage. Freeze-drying (*26*, *83*) and spray-drying (*25*, *84*, *85*) are the most commonly used for drying of the particles.

Multilayer emulsions may have potential applications in the food or cosmetic industry, as this process offers advantages such as protecting emulsion droplets from oxidation or lipid aggregation, controlling or releasing active materials, and improving stability against environmental agents due to the thicker interfacial layers (*25*, *86*). The electrostatic deposition method is, therefore, a versatile tool for modulating the functional performance of emulsion-based delivery systems by altering their interfacial properties (*87*). Another important advantage of multilayer emulsions is the protection against thermal stress promoted by drying methods.

Despite its potential applications and advantages, it is important to mention that there are certain disadvantages that may limit the widespread commercialization of multilayer emulsions. It is necessary to have precise control over the system composition and preparation procedures to avoid droplet bridging, depletion and other effects. Consequently, this type of system is more expensive and difficult to set up, but the potential benefits (enhanced functionality or extended life) can outweigh the costs in certain applications (*77*).

Stability. Noello *et al*. (*84*) produced chia oil microparticles using both emulsions stabilized by whey protein concentrate and pectin through the electrostatic layer-by-layer deposition and emulsions prepared only with whey protein concentrate. The microparticles were dried by spray-drying and showed higher oxidative stability than the pure oil. The emulsions obtained by electrostatic deposition had smaller droplet diameters and greater stability than emulsions produced only with whey.

Fioramonti *et al.* (*86*) investigated the influence of acid pH and sodium alginate concentration on the flaxseed oil properties in water emulsions stabilized by whey protein isolate. The same authors conducted another study about dehydration of microparticles (*83*). Freeze-drying was chosen due to the bioactive compounds present in flaxseed oil. However, the techniques applied to obtain microparticles by ultrasonic emulsification and freeze-drying contributed significantly to the oil oxidation, as high input energies during ultrasonic emulsification promoted an increase in lipid oxidation rates, with the consequent formation of highly reactive peroxides, whose concentration was beyond the limit allowed for food matrices. Again, Fioramonti *et al.* (*85*) evaluated the influence of homogenization pressure and spray-drying. The experiments showed that it was possible to transform a highly oxidizable ingredient of liquid phase into a solid, easy-to-handle powder with peroxide values within the permitted range for food.

Applications. Carvalho *et al.* (*25*) microencapsulated green coffee oil using emulsions stabilized by lecithin and chitosan through electrostatic layer-by-layer deposition followed by spray-drying. Thus obtained microparticles exhibited better results for sun protection and oxidative stability than the free oil, with potential applications in cosmetics and personal care. Julio *et al.* (*26*) did the physicochemical characterization of chia oil microparticles produced from mono- and bi-layer emulsions using modified sunflower lecithin, chitosan and maltodextrin as matrix. The two-layer microparticles with modified sunflower lecithin were effective in protecting the chia oil against oxidative deterioration, being an adequate delivery system for the ω-3 fatty acids in this oil for the functional food application.

### Chemical methods

The chemical methods differ from the physicochemical methods in that a reaction occurs, the most common being polymerization, forming a wall with appropriate mechanical and chemical properties and increasing the reliability of the process. Of the studied methods, this approach is the least used, because the studies require better optimizations such that the polymerization reaction does not interfere with the properties of the bioactive compound of interest. Furthermore, it is a laborious process that depends on the conditions of reaction, *e.g.* type of emulsifier, stirring speed, core/matrix mass ratio, pH value, reaction temperature and hydrophobic core surface.

### Interfacial/*in situ* polymerization

Principles. Microencapsulation of an active material by interfacial polymerization involves an oil-soluble phase dispersion in a continuous aqueous phase or an aqueous phase dispersion in an organic phase, depending on the solubility of the encapsulated core substance and the conditions for the precipitation of polymeric materials at the drop interface. Each phase contains a specific dissolved monomer suitable to react with the other monomer present in the other phase. The dispersed phase acts as a good solvent for the monomers, but it also serves as a non-solvent for the polymer produced in the reaction. Therefore, during polymerization, the system is composed of three mutually immiscible phases. Once the various monomers are added in drops to the system, reactions take place at the interface, resulting in the formation of insoluble oligomers in each drop with the tendency to precipitate at the interface to form a primary shell around the drop. The formed polymer is not soluble in the dispersed or continuous phase and precipitates (*88*, *89*).

The interfacial polymerization technique has potential advantages, including possible control of microcapsule average size and shell thickness, high load of the active compound, versatile and stable mechanical and chemical properties of the shell, low cost, easy scale-up, simplicity and reliability of the process (*90*, *91*). On the other hand, there are also some factors that limit the application of this technique, such as the production of a large oil-water interface, where proteins or enzymes are prone to inactivation, altering the biological activities of proteins during the polymerization reaction (*8*). Another negative point is that the resulting microcapsules usually have certain unreacted shell monomers, which can react with the core material and potentially cause its deactivation or other unwanted consequences (*92*).

**Fig. S4:**
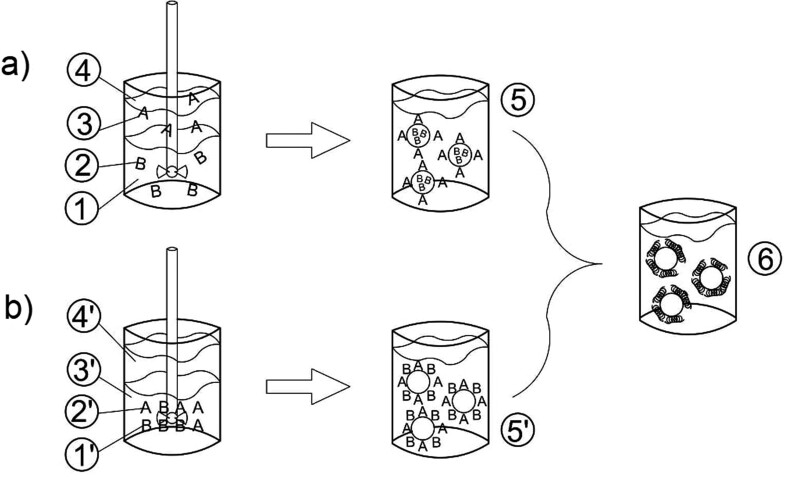
Microencapsulation by polymerization: a) interfacial, where: 1=aqueous solution (hydrophilic phase), 2=monomer B, 3=monomer A, 4=oil (lipophilic phase), 5=diffusion of monomers to the interface, and 6=polymerization reaction and matrix formation, and b) *in situ*, where 1'=monomer B, 2'=monomer A, 3'=aqueous solution (hydrophilic phase), 4'=oil (lipophilic phase), 5'=dissolving the monomers in the continuous phase, and 6=polymerization reaction and matrix formation

*In situ* polymerization is similar to interfacial polymerization; however, the reagents involved in the synthesis of the active matrix are obtained from both dispersed and continuous phases. During *in situ* polymerization (Fig. S4), oil-in-water or water-in-oil emulsions are first produced under vigorous stirring or sonication of a biphasic liquid. The monomers and initiators used to build the capsule matrix are dissolved in the dispersed or continuous phase. As the polymer synthesized from the monomers is insoluble in the emulsion, polymerization usually occurs on the core material droplet surface, and the resulting polymer accumulates on the droplet surface, generating microcapsules with the desired core material (*92*, *93*). The controlled deposition and polymer precipitation occur at the interface using precipitants, or a change in pH, temperature or solvent quality (*93*).

*In situ* polymerization requires longer reaction times than other encapsulation techniques. However, this technique offers some advantages, such as low cost, ease of industrial manufacturing and simplicity of the procedure (*94*). It should be noted that the microcapsule preparation by *in situ* polymerization depends not only on the core materials and the encapsulating matrix, but also on the reaction conditions, *i.e.* the type of emulsifier, agitation speed, core/matrix mass ratio, pH value, reaction temperature and hydrophobic surface of the core (*95*, *96*).

Stability. Moser *et al.* (*97*) studied the formation of microcapsules produced by the interfacial polymerization of chickpea protein and pectin in buriti oil emulsions and dried by spray-drying. The microencapsulation and drying did not influence the lipid oxidation, showing that the emulsions produced by interfacial polymerization protected the oil from the high temperatures of spray-drying. Suryanarayana *et al.* (*98*) prepared linseed oil microcapsules coated by *in situ* polymerization of urea-formaldehyde resin. These microcapsules were incorporated into a paint formulation. The mechanical stability was studied and the microcapsules showed sufficient strength to withstand the shear generated during mixing and application of the paint.

Applications. Bagle *et al.* (*27*) used the microencapsulated neem oil as a biopesticide by *in situ* polymerization of phenol formaldehyde in an oil-in-water emulsion. The synthesized microcapsules also showed good thermal stability, necessary for long-term core preservation. Slow release of core material was observed at 6 h (about 30%). Therefore, neem oil as a biopesticide can be microencapsulated with a phenol formaldehyde polymer for better preservation and efficient controlled release applications.

## CONCLUSIONS

In this review, several techniques commonly used in the microencapsulation of vegetable oil were presented. The advantages and disadvantages of each technique were discussed in order to assist in the choice of an appropriate approach for the encapsulation of the vegetable oil. Because the needs are different for the food, cosmetic, personal care and pharmaceutical industry, it is useful to have all these techniques available for use. From the publications in the literature, it is clear that this field is still growing and evolving, and we expect further developments and improvements in the future.
